# ERK-associated changes in E2F4 phosphorylation, localization and transcriptional activity during mitogenic stimulation in human intestinal epithelial crypt cells

**DOI:** 10.1186/1471-2121-14-33

**Published:** 2013-08-06

**Authors:** Marie-Christine Paquin, Sébastien Cagnol, Julie C Carrier, Caroline Leblanc, Nathalie Rivard

**Affiliations:** 1 Département d’Anatomie et Biologie Cellulaire, Cancer Research Pavillon, Faculté de Médecine et des Sciences de la Santé, Université de Sherbrooke, 3201, Jean-Mignault, Sherbrooke, J1E4K8, QC, Canada

**Keywords:** E2F, Intestinal epithelium, Proliferation, Colorectal cancer, Cell cycle, GSK3, ERK, EGF

## Abstract

**Background:**

The transcription factor E2F4 controls proliferation of normal and cancerous intestinal epithelial cells. E2F4 localization in normal human intestinal epithelial cells (HIEC) is cell cycle-dependent, being cytoplasmic in quiescent differentiated cells but nuclear in proliferative cells. However, the intracellular signaling mechanisms regulating such E2F4 localization remain unknown.

**Results:**

Treatment of quiescent HIEC with serum induced ERK1/2 activation, E2F4 phosphorylation, E2F4 nuclear translocation and G1/S phase transition while inhibition of MEK/ERK signaling by U0126 prevented these events. Stimulation of HIEC with epidermal growth factor (EGF) also led to the activation of ERK1/2 but, in contrast to serum or lysophosphatidic acid (LPA), EGF failed to induce E2F4 phosphorylation, E2F4 nuclear translocation and G1/S phase transition. Furthermore, Akt and GSK3β phosphorylation levels were markedly enhanced in serum- or LPA-stimulated HIEC but not by EGF. Importantly, E2F4 phosphorylation, E2F4 nuclear translocation and G1/S phase transition were all observed in response to EGF when GSK3 activity was concomitantly inhibited by SB216763. Finally, E2F4 was found to be overexpressed, phosphorylated and nuclear localized in epithelial cells from human colorectal adenomas exhibiting mutations in *APC* and *KRAS* or *BRAF* genes, known to deregulate GSK3/β-catenin and MEK/ERK signaling, respectively.

**Conclusions:**

The present results indicate that MEK/ERK activation and GSK3 inhibition are both required for E2F4 phosphorylation as well as its nuclear translocation and S phase entry in HIEC. This finding suggests that dysregulated E2F4 nuclear localization may be an instigating event leading to hyperproliferation and hence, of tumor initiation and promotion in the colon and rectum.

## Background

E2F transcription factors control exit from cell quiescence and progression through G1 and S phase entry [1,2]. In mammalian cells, E2F consists of a family of related proteins that includes eight members which pair with a heterodimeric partner (DP1 or DP2) [2]. The first three family members, E2F1, 2 and 3a, are primarily considered as transcriptional activators. Their functions are partially redundant and simultaneous abrogation of the three is lethal at an early embryonic stage [3]. Unlike E2F1, 2 or 3a, E2F4 and 5 have mostly been described as transcriptional repressors, at least in fibroblasts [2]. However, in rapidly renewing tissues such as bone marrow, skin and digestive tract where E2F4 is predominantly expressed [4], this latter E2F family member appears to act as an activator of both transcription and cell cycle progression. Indeed, in mice, deletion of the *E2F4* gene causes a reduction in the number of erythrocytes due to impaired proliferation of progenitors in bone marrow [5]. In skin, overexpression of E2F4 results in hyperproliferation of basal keratinocytes and induces hyperplasia [6]. In the small intestine, loss of *E2F4* results in a significant decline in proliferative zones (crypts) and a shortening of intestinal villi [4]. In contrast, loss of *E2F1* expression does not affect intestinal development or homeostasis [7]. In addition, E2F4 is also strongly and preferentially expressed in proliferative zones of embryonic mouse intestine [8] and human fetal intestinal epithelium [9]. Finally and more importantly, inhibition of E2F4 expression by RNA interference in normal and cancerous intestinal epithelial cells reveals that E2F4 is necessary for S-phase entry and proliferation [10].

Several reports indicate that subcellular localization of E2F4 controls its transcriptional activity [11-14]. Accordingly, we have recently shown that the cellular localization of E2F4 is cell cycle-dependent in normal intestinal epithelial cells. Indeed, in contrast to E2F1, which constitutively resides in the nucleus throughout the cell cycle, E2F4 is mostly distributed in the cytoplasm of quiescent intestinal crypt cells and translocates into the nucleus upon serum stimulation [9]. Hence, this suggests that cytoplasmic sequestration or nuclear export of E2F4 may provide a means to control its transcriptional activity. However, the intracellular mechanisms by which serum growth factors induce E2F4 nuclear translocation remain to be identified.

Herein, we show that activation of MEK/ERK signaling by serum is required for E2F4 nuclear translocation as well as for G1/S phase transition of human non immortalized intestinal epithelial crypt cells (HIEC) in culture. Our results demonstrate that ERK1/2 directly and rapidly phosphorylates E2F4 following serum stimulation and is correlated with its increased transcriptional activity and S phase entry. However, although epidermal growth factor (EGF) treatment resulted in rapid activation of ERK1/2, it was not sufficient to promote E2F4 translocation into the nucleus or G1/S phase transition in HIEC. Additional GSK3 inhibition was required for these events to occur in presence of EGF. Finally, we show that E2F4 is overexpressed, phosphorylated and localized in the nucleus of epithelial cells from colorectal adenomas exhibiting *APC* and *KRAS* or *BRAF* mutations. Taken together, our results emphasize the importance of regulating E2F4 localization for proliferation in normal human intestinal epithelial cells as well as in intestinal tumors.

## Results

### MEK/ERK pathway is required for E2F4 nuclear translocation and G1/S phase transition of HIEC

**Figure 1:**
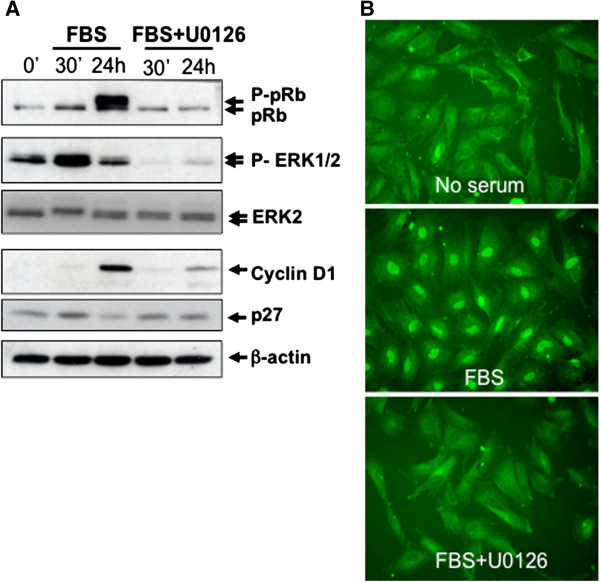
**MEK/ERK pathway is required for E2F4 nuclear translocation and G1/S phase transition in HIEC. A.** Subconfluent HIEC were serum-deprived for 36 h, treated or not (DMSO) during 10 min with 20 μM U0126 and then stimulated during 30 min or 24 h with 5% FBS. Thereafter, cells were lysed and proteins were analyzed by SDS-PAGE for Western blot analysis of the expression of ERK2, pRb, cyclin D1, p27, phosphorylated ERK1/2 and β-actin. **B.** HIEC grown on coverslips were serum-deprived during 36 h and then stimulated with 5% FBS with or without 20 μM U0126 (or DMSO). After 24 h, cells were fixed using 3% paraformaldehyde, permeabilized and analyzed by immunofluorescence for E2F4 expression.

We have previously shown that E2F4 is required for proper expression of many cell cycle regulatory proteins controlling G1/S phase transition and for proliferation of normal human intestinal epithelial cells (HIEC) [10]. In contrast to E2F1, which is constitutively localized in the nucleus, E2F4 has a diffuse cytoplasmic localization in quiescent HIEC and a nuclear localization in proliferative cells suggesting that its localization is regulated by signaling pathways activated by mitogens [9]. In light of the above, we analyzed the signaling pathways that could be involved in serum-induced E2F4 nuclear translocation and G1/S phase transition in HIEC. We first verified the involvement of the MEK/ERK pathway given that we had previously demonstrated that sustained activation of ERK1/2 is required for intestinal epithelial cells to enter S-phase [15]. Among physiological events relevant for G1/S phase transition, there is the phosphorylation of the retinoblastoma gene product pRb by cyclin D/Cdk4,6 and cyclin E/Cdk2 complexes, which causes the release and activation of E2F/DP transcription factors [1]. E2F4 localization and hyperphosphorylation of pRb were therefore analyzed following treatment of HIEC with serum in absence or presence of U0126, a potent inhibitor of MEK1/2 (the upstream activators of ERK1/2). As expected, addition of 20 μM U0126 to HIEC potently inhibited serum-induced ERK1/2 phosphorylation (Figure 1A) without affecting phosphorylation of other signaling kinases such as ERK5 and AKT (Additional file 1: Figure S1). Of note, the stimulatory effect of serum on cyclin D1 expression, p27 down-regulation and pRb hyperphosphorylation was also abolished by U0126. Furthermore, U0126 treatment totally prevented nuclear translocation of E2F4 in response to serum (Figure 1B).

### E2F4 is phosphorylated by ERK upon serum stimulation

**Figure 2:**
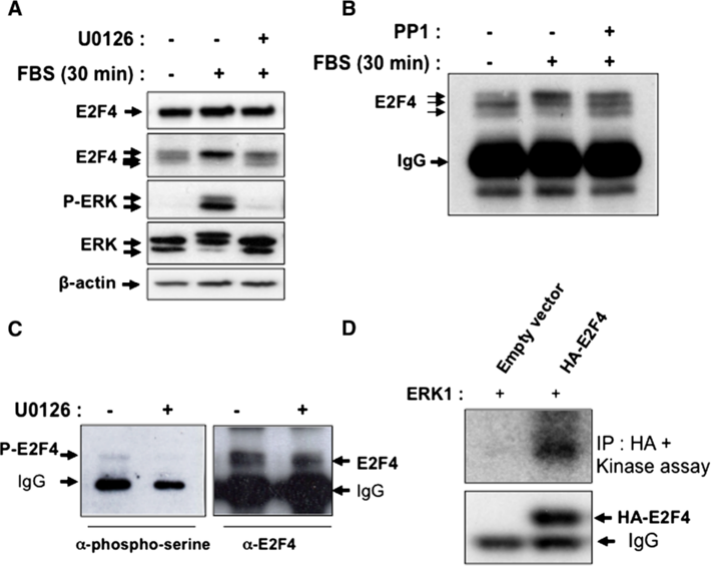
**E2F4 is phosphorylated by ERK kinases. A.** Subconfluent HIEC were serum-deprived for 36 h, treated or not (DMSO) during 10 min with 20 μM U0126 and then stimulated during 30 min with 5% FBS. Thereafter, cells were lysed and proteins were analyzed by SDS-PAGE (on 7.5% or 12% acrylamide gels) for Western blot analysis of the expression of phosphorylated ERK1/2, total ERK1/2, E2F4 and β-actin. **B.** E2F4 was immunoprecipitated from subconfluent serum-deprived and 30 min serum-stimulated HIEC. Beads containing E2F4 immune complexes were incubated with PP1 phosphatase for 30 min prior to Western blot analysis for E2F4 expression. **C.** E2F4 was immunoprecipitated from HIEC stimulated during 30 min with FBS in presence or absence of 20 μM U0126. E2F4 immune complexes were analyzed by SDS-PAGE for Western blot analysis with antibodies recognizing phosphorylated serine or E2F4. **D.** 293T cells were transfected with pCDNA3.1 containing or not HA-E2F4. After 48 h, cells were lysed and immunoprecipitated with anti-HA antibody. Kinase assays were performed by incubating beads containing HA-E2F4 immune complexes with active recombinant ERK1 for 5 min. Radiolabeled proteins were separated on SDS-PAGE and autoradiographed or analyzed by Western blot for the expression of E2F4.

Western blot analysis of E2F4 revealed that 30 min serum stimulation with or without U0126 did not affect the total expression levels of E2F4 (Figure 2A, upper first panel), even after 24 h stimulation (data not shown). However, when using higher-resolution gels, three major bands of approximately 60–63 kDa were detected in serum-deprived HIEC, whereas only one band with a lower electrophoretic mobility was observed in serum-stimulated cells after 30 min (Figure 2A, arrows). Of note, treatment with U0126 abolished ERK phosphorylation and markedly reduced the expression of this latter prominent band (Figure 2A). Similar results were obtained when we used the more specific and potent MEK inhibitor PD184352 (Additional file 2: Figure S2). We therefore investigated whether E2F4 phosphorylation could be responsible for this occurrence. E2F4 was immunoprecipitated from serum-deprived or serum-stimulated HIEC. Beads containing E2F4 immune complexes were then incubated with the serine/threonine phosphatase PP1 in order to dephosphorylate serine/threonine residues on E2F4. As shown in Figure 2B, immunoprecipitated E2F4 exhibited three bands in control HIEC, in contrast to one prominent band in serum-stimulated cells. Of interest, treatment of E2F4 immunoprecipitates from serum-stimulated cells with the PP1 phosphatase modified the electrophoretic profile of E2F4, reducing the amount of the slower migrating form of E2F4. Moreover, the use of antibodies recognizing phosphorylated serine revealed that E2F4 was phosphorylated on serine residue(s) upon serum stimulation (Figure 2C). Of note, the levels of phosphorylated serine residues in immunoprecipitated E2F4 were not completely reduced by U0126 treatment, suggesting that E2F4 could also be phosphorylated in absence of serum and ERK activation in quiescent HIEC as we previously observed [9]. Kinase assays with active recombinant ERK1 confirmed that ERK1 strongly phosphorylated immunoprecipitated HA-tagged E2F4 *in vitro* (Figure 2D). These results clearly indicate that E2F4 is phosphorylated on serine residue(s) in response to serum in a MEK-dependent manner. The data also suggest ERK1/2 as candidate kinases.

### Phosphorylation of E2F4 on serines 244 and 384 promotes its transcriptional activity

**Figure 3:**
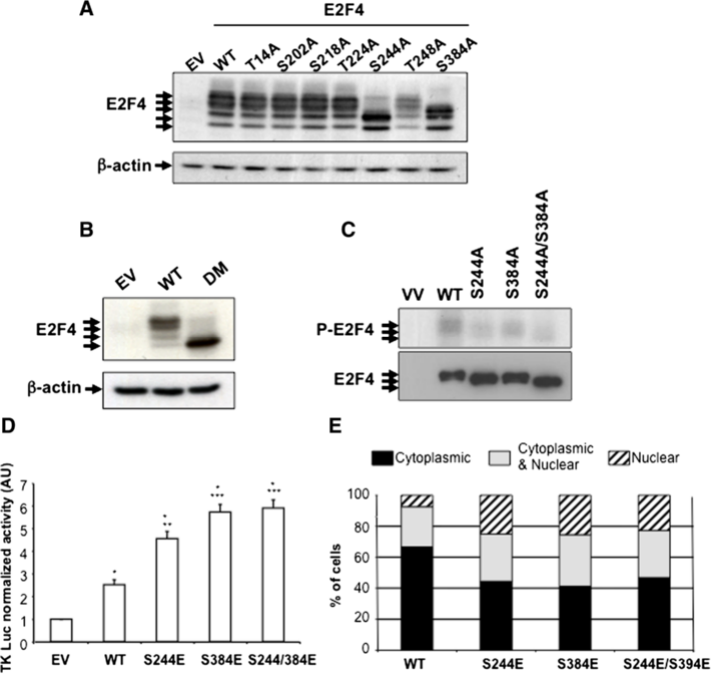
**Phosphorylation of E2F4 on serines 244 and 384 promotes its transcriptional activity. A.** and **B.** 293T cells were transfected with pCDNA3.1 empty vector (EV) or encoded for HA-tagged- human wild-type (WT) E2F4 or E2F4 mutants as indicated. After 48 h, cell lysates were analyzed for the expression of E2F4 proteins. **C.** 293T cells were transfected with EV or encoding for HA-E2F4 WT, HA-E2F4 S244A, HA-E2F4 S384A or HA-E2F4 S244A/S384A. After 48 h, cells were lysed and immunoprecipitated with anti-HA antibody. Kinase assays were performed by incubating beads containing HA-E2F4 immune complexes with recombinant ERK1 for 5 min. Radiolabeled proteins were separated on SDS-PAGE and autoradiographed or analyzed for the expression of E2F4. **D.** 293T cells were co-transfected with DP2, *thymidine kinase* luciferase reporter with either empty vector, HA-wtE2F4, HA-E2F4 S244E, HA-E2F4 S384E or HA-E2F4 S244/S384E. pRL-SV40 Renilla luciferase reporter control vector was also co-transfected. Forty-eight hours following transfection, luciferase activity was quantified and normalized using the Renilla reporter, with the empty vector condition set at 1. A representative experiment of three experiments is shown. *Significantly different from control at *p* < 0.001, **Significantly different from WT at *p* < 0.002, ***Significantly different from WT at *p* < 0.001 (Student’s *t* test). **E.** HIEC grown on coverslips were transfected with either empty vector, HA-wtE2F4, HA-E2F4 S244E, HA-E2F4 S384E or HA-E2F4 S244/S384E. After 48 h, cells were analyzed by immunofluorescence for subcellular localization of HA-tagged E2F4 forms. Total cell number was determined using DAPI staining and cells exhibiting E2F4 forms into the nucleus or into the cytoplasm or in both compartments were counted. The percentage of cells exhibiting HA staining into the nucleus, into the cytoplasm or in both compartments was calculated and shown in the graph. Representative experiment is shown.

We identified seven putative ERK phosphorylation sites followed by a proline residue in E2F4 human sequence: T14, S202, S218, T224, S244, T248 and S384. Each of these specific serine/threonine residues was mutated into alanine. As shown in Figure 3A, mutation of serines 244 and 384 resulted in modification of the E2F4 electrophoretic profile in 293T cells, decreasing the amount of the slower migrating forms of E2F4. Of note, these slower migrating forms almost completely disappeared when both serines were mutated into alanine (Figure 3B). Accordingly, the S244A, S384A and the S244A/S384A mutants were much less phosphorylated by recombinant ERK1 in *in vitro* kinase assays (Figure 3C). Finally, the effect of E2F4 phosphorylation on E2F4 site-dependent transcription was measured on the *thymidine kinase* promoter, which represents the physiological E2F target gene [1,2]. Mutation of each of these serines into phosphomimetic sites, namely S244E, S384E and S244E/S384E, significantly increased the transcriptional activity of E2F4 (Figure 3D), confirming the involvement of the phosphorylation of these residues in the control of E2F4 transcriptional activity. To verify if mutations of serines 244 and 384 also alter the localization of E2F4, the S244A, S384A and the S244A/S384A E2F4 mutants were transiently expressed in HIEC and analyzed for their subcellular localization. As shown in Figure 3E, when overexpressed, wild-type E2F4 was mainly found into the cytoplasm but also in a minor proportion into the nucleus. By contrast, all the phosphomimetic mutants were consistently less expressed into the cytoplasm and more detected into the nucleus, correlating with their increased transcriptional activity (Figure 3D). Nevertheless, the observation of some cytoplasmic staining for the phospho-mimetic mutants indicate that other mechanisms than phosphorylation might also be involved in the control E2F4 nuclear translocation.

### EGF neither induces E2F4 phosphorylation and nuclear translocation nor G1/S phase transition in HIEC

**Figure 4:**
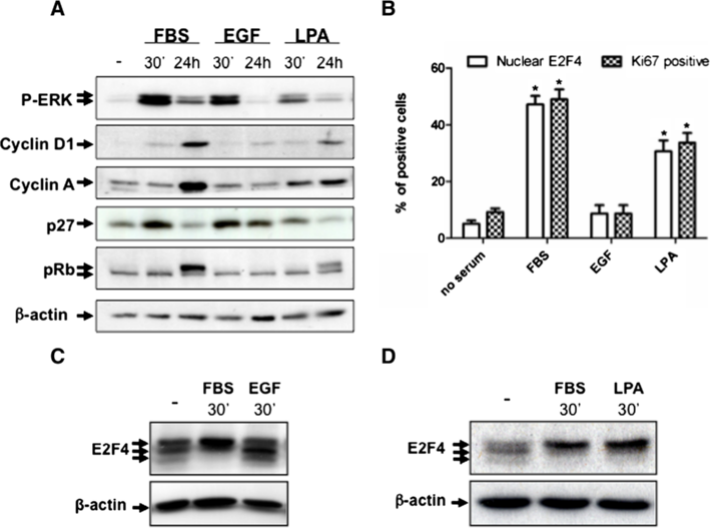
**EGF neither induces E2F4 phosphorylation and nuclear translocation nor G1/S phase transition in HIEC. A.** Subconfluent HIEC were serum-deprived during 36 h, stimulated with 5% FBS or 100 ng/ml EGF or 10 μM LPA for 30 min, 4 h or 24 h. Equal amounts of whole cell lysates were separated by SDS-PAGE, and proteins were analyzed by Western blotting with specific antibodies against phosphorylated ERK1/2, pRb, cyclin D1, cyclin A, p27 and β-actin. **B.** Cells were also fixed after 24 h stimulation with 3% paraformaldehyde in PBS and permeabilized with 0.1% Triton X-100 for subsequent immunofluorescence staining of E2F4 and Ki67. Cells with nuclear E2F4 and Ki67 were counted in 10 fields. Total cell number was determined using DAPI staining. Ratio of nuclear E2F4 expressing cells and Ki67 positive cells before/after serum, EGF or LPA stimulation are shown. Of note, each cell exhibiting nuclear E2F4 was positive for Ki67 staining. Results are the mean ± SEM of an experiment representative of 3. * Significant at *p* < 0.0001 compared to control cells (no serum) (Student’s *t* test). **C** and **D.** HIEC were serum-deprived during 36 h, stimulated with 5% FBS or 100 ng/ml EGF or 10 μM LPA for 30 min. Cell lysates were separated by 7.5% SDS-PAGE and proteins were analyzed by Western blotting with specific antibodies against E2F4 and β-actin.

To better understand the mechanisms controlling subcellular E2F4 localization during G1/S phase transition in HIEC, we analyzed the impact of growth factors such as EGF and lysophosphatidic acid (LPA) on these events. EGF is a classical growth factor that can activate many intracellular signaling cascades including the ERK1/2 MAP Kinase cascade [16,17]. In addition, we and others have previously demonstrated that EGF induces proli-feration of rat immortalized intestinal epithelial cells in a MEK-dependent manner [17,18]. In the present series involving human intestinal epithelial cells, although EGF treatment resulted in a rapid and sustained activation of ERK1/2 (at least > 4 h) and in a modest induction of cyclin D1, it was not sufficient to mimic serum-induced cyclin A expression, p27 down-regulation and pRb hyperphosphorylation (Figure 4A). Importantly, in contrast to serum, EGF stimulation did not trigger E2F4 translocation into the nucleus and no Ki67 staining was observed (Figure 4B). Of note, Western blot analysis with an anti-E2F4 antibody revealed three major bands in EGF-stimulated HIEC similar to controls, whereas E2F4 displayed a typical phosphorylated low electrophoretic mobility in serum-stimulated cells (Figure 4C). Neither pRb hyperphosphorylation nor Ki67 staining was observed in the presence of lower and higher EGF concentrations ranging from 2 ng/ml to 1 ug/ml (data not shown), thus eliminating the likelihood of overactivation or underactivation of EGF receptors. Furthermore, HIEC proliferation was not enhanced even after several days of incubation with EGF (data not shown). Hence, these results indicate that EGF by itself is not able to trigger S phase entry and proliferation of normal human non immortalized intestinal epithelial crypt cells.

We next investigated the impact of LPA, a biologically active lysophospholipid that mediates a plethora of cellular effects, including cell proliferation of many cell types such as fibroblasts, breast cancer cells, mesangial cells, vascular smooth muscle cells, neuronal cells and human colorectal cancer cells [19]. As shown in Figure 4A and B, similarly to serum stimulation, LPA clearly induced ERK1/2 phos-phorylation and HIEC cell cycle entry as visualized by pRb hyperphosphorylation, cyclin D1 expression, cyclin A expression, decreased p27 expression, E2F4 nuclear translocation and Ki67 staining. Western blot analysis with anti-E2F4 antibody also indicated that LPA induced E2F4 phosphorylation to a level comparable to that observed with serum (Figure 4D). Thus, in contrast to EGF, LPA is sufficient to promote phosphorylation and nuclear translocation of E2F4 as well as S-phase entry demonstrating that LPA is an important contributing serum growth factor for HIEC.

### GSK3 inhibition is required for phosphorylation and nuclear translocation of E2F4 as well as G1/S phase transition in HIEC

**Figure 5:**
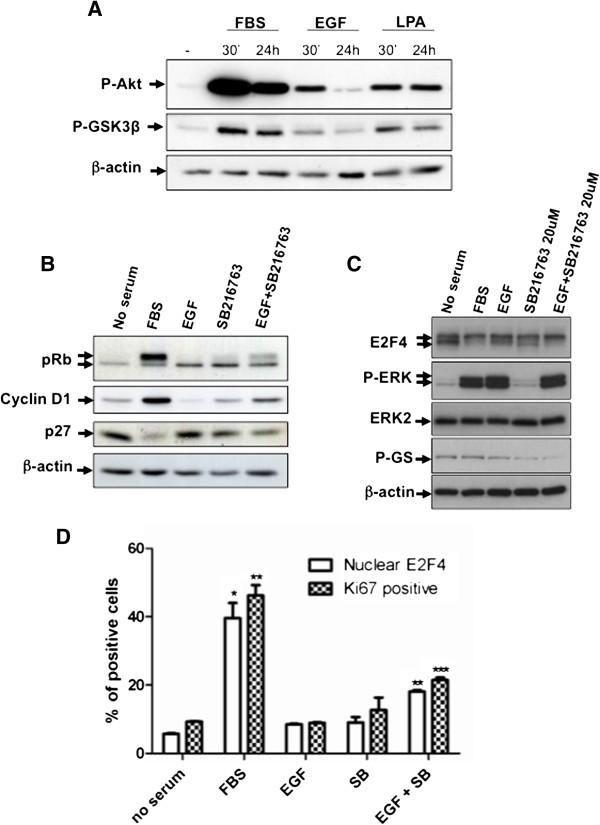
**GSK3 inhibition is required for phosphorylation and nuclear translocation of E2F4 as well as G1/S phase entry of HIEC. A.** Subconfluent HIEC were serum-deprived during 36 h and then stimulated with 5% FBS or 100 ng/ml EGF or 10 μM LPA for 30 min and 24 h. Equal amounts of whole cell lysates were analyzed by Western blotting with specific antibodies against phosphorylated Akt, phosphorylated GSK3β and β-actin. **B.** Subconfluent HIEC were serum-deprived during for 36 h, stimulated with 5% FBS or 100 ng/ml EGF in presence or absence of 20 μM SB216763 (or DMSO) for 24 h. Equal amounts of whole cell lysates were separated by SDS-PAGE, and proteins were analyzed by Western blotting with specific antibodies against pRb, cyclin D1, p27 and β-actin. **C.** HIEC were serum-deprived during 36 h and then stimulated with 5% FBS or 100 ng/ml EGF for 30 min with or without prior 10-min 20 μM SB216763 (or DMSO) treatment. Cell lysates were analyzed by Western blotting with specific antibodies against E2F4, ERK2, phosphorylated ERK1/2, phosphorylated glycogen synthase (GS) and β-actin. **D.** Cells were also fixed after 24 h stimulation and permeabilized with 0.1% Triton X-100 for subsequent immunofluorescence staining of E2F4 and Ki67. Cells with nuclear E2F4 and positive for Ki67 staining were counted in 10 fields. Total cell number was determined using DAPI staining. Ratio of nuclear E2F4 expressing cells and Ki67 positive cells before/after serum or EGF +/− SB216763 (SB) stimulations are shown. Of note, each cell exhibiting nuclear E2F4 was positive for Ki67 staining. Results are the mean ± SEM of 2 separate experiments. * Significant at *p* < 0.05. ** Significant at *p* < 0.01. *** Significant at *p* < 0.005 compared to control cells (no serum) (Student’s *t* test).

In order to elucidate why EGF, in contrast to LPA and serum, failed to induce G1/S phase transition in HIEC, we analyzed the phosphorylation levels of GSK3β. GSK3β is a constitutively active serine/threonine kinase featured in two signaling pathways that are of particular importance for intestinal epithelial cell proliferation and colorectal cancer: the Wnt/β-catenin pathway and the phosphatidylinositol 3-kinase (PI3K)/Akt pathway [16,20,21]. Indeed, Akt phosphorylates GSK3β on serine 9, leading to inhibition of its constitutive kinase activity. GSK3β is also a component of Wnt signaling, which is thought to block GSK3-mediated β-catenin phosphorylation, leading to the accumulation and nuclear translocation of β-catenin [22]. As shown in Figure 5A, serum markedly induced phosphorylation levels of Akt and GSK3β after both 30 min and 24 h stimulation. By contrast, stimulation of HIEC with EGF only transiently increased phosphorylation of Akt and GSK3β. Interestingly, LPA induced a more sustained Akt and GSK3β phosphorylation (Figure 5A).

These results prompted us to verify whether GSK3 inhibition was necessary for E2F4 phosphorylation and nuclear translocation as well as G1/S phase transition in HIEC. To test this hypothesis, a specific GSK3 inhibitor (SB216763) was used. HIEC were serum-starved for 36 h, pre-treated 10 min with SB216763 and then stimulated for 30 min or 24 h with EGF. As shown in Figure 5B, a 24 h-treatment of HIEC with EGF or GSK3 inhibitor alone was not sufficient to significantly induce expression of cyclin D1, p27 down-regulation or pRb hyperphosphorylation in contrast to serum. However, significant pRb hyperphosphorylation as well as cyclin D1 expression and p27 down-regulation were observed in presence of EGF and SB216763. Furthermore, inhibition of GSK3 significantly reduced phosphorylation of glycogen synthase (GS) but enhanced the capacity of EGF to promote E2F4 phosphorylation (Figure 5C). Accordingly, in presence of SB216763, EGF significantly induced E2F4 nuclear translocation and Ki67 staining (Figure 5D).

### E2F4 is overexpressed, phosphorylated and nuclear localized in human colorectal adenomas

**Figure 6:**
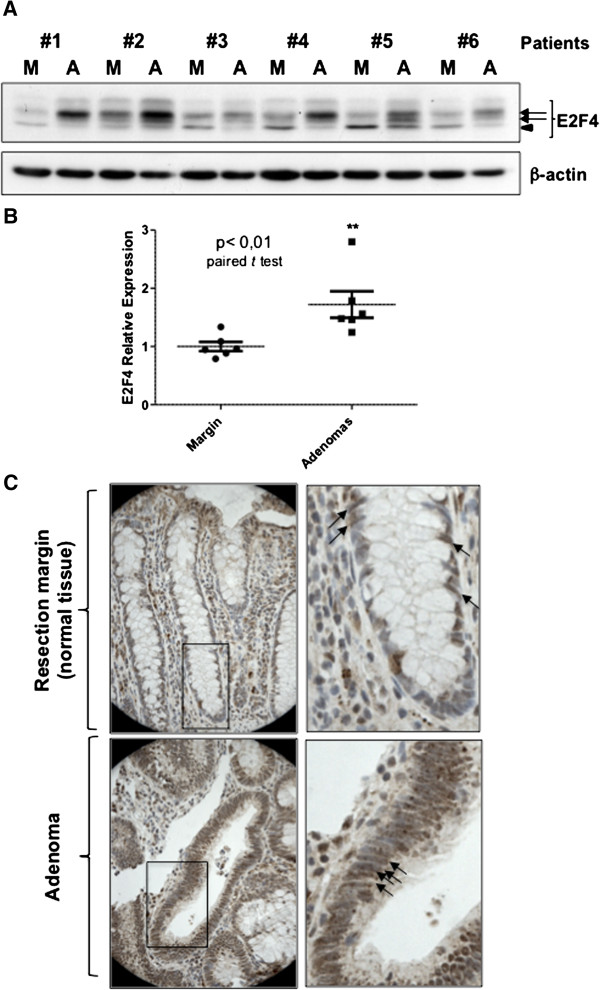
**E2F4 is overexpressed, phosphorylated and nuclear localized in epithelial cells of human colorectal adenomas. A.** E2F4 and β-actin protein levels were determined in six paired specimens of advanced adenomas by Western blot (resection margins (M) and primary tumors). **B.** E2F4 expression levels were normalized to β-actin levels. E2F4 expression levels were also normalized to a mean of E2F4 expression levels in every resection margins, resulting in a fold induction value. E2F4 protein contents in adenoma tissues relative to their corresponding normal samples were analyzed by paired *t*-test. **, Significant at p < 0.01. **C.** Representative immunohistochemistry of E2F4 from frozen tissue sections of resection margin and corresponding adenoma. Bars: 100 μm. Higher magnifications are shown on the left panels.

Both MEK/ERK and GSK3 signaling pathways are thought to be affected in early stages of colorectal cancer formation due to frequent mutations in *KRAS*/*BRAF* and *APC* genes respectively [23]. We therefore verified the protein status of E2F4 in human colorectal adenomas. As shown in Figure 6A and B, adenomas displayed significantly higher expression levels of E2F4 in comparison to their corresponding benign epithelium (margin). More importantly, all normal specimens analyzed (resection margins, M) presented hypophosphorylated forms of E2F4 (arrowhead) whereas all adenoma samples (A) exhibited hyperphosphorylated forms of E2F4 (arrows) (Figure 6B). Furthermore, immunohistochemical analysis demonstrated that E2F4 protein was especially overexpressed in the nucleus of all epithelial cells in colorectal adenomas (Figure 6C, lower panels, arrows) while only localized in the nucleus of certain cells along the colonic crypt, presumably in proliferative cells (Figure 6C, right panel, arrows) as previously demonstrated [9]. Of note, all of the adenomas analyzed exhibited *APC* inactivating mutations (exon 15) in combination with *KRAS* (G12D, G13D, Q61H) or *BRAF* (V600E) activating mutations. Hence, these results emphasize the close correlation between the phosphorylation of E2F4 and its nuclear localization.

## Discussion

The intestinal epithelium is the most vigorously self-renewing tissue in adult mammals. Perturbations of normal tissue homeostasis attributable to genetic lesions or environmental insults can lead to hyperproliferative diseases of the intestinal tract such as cancer [24]. Intestinal epithelial cell regulation has been studied extensively and has revealed canonical Wnt/β-catenin, KRAS/MAPK and PI3K/Akt signaling pathways as key regulators of cell division and differentiation [16,18,20]. Normal cell division is a tightly controlled process that only allows cells to divide in a timely and restricted manner. As such, E2F transcription factors control cell division by activating the transcription of numerous genes involved in G1 and S phases [1,2]. Many previous analyses of E2F proteins in intact intestinal epithelium or in cultured crypt cells have demonstrated that nuclear E2F4 may be determinant in the control of proliferation. Indeed, in mice, deletion of *E2F4* gene resulted in a significant decline in proliferative zones (crypts) and a shortening of intestinal villi [4]. Accordingly, double staining experiments in intact human intestine revealed that crypt epithelial cells expressing high levels of nuclear E2F4, were all positive for Ki67 and cyclin A [9,10]. In this respect, decreased expression of E2F4 by RNA interference reduced the proliferation rate of normal intestinal epithelial crypt cells and colorectal cancer cells in culture [10]. Interestingly, in contrast to E2F1, which resides constitutively in the nucleus throughout the cell cycle, E2F4 protein is mostly distributed in the cytoplasm of quiescent cells and translocates into the nucleus upon serum stimulation [9]. Taken together, these results suggest that the nuclear translocation of E2F4 may represent a critical step in promoting G1/S transition in intestinal epithelial crypt cells.

In the present study, we show that activation of the MEK/ERK pathway by serum is required for E2F4 nuclear translocation as well as for G1/S phase transition of human intestinal epithelial crypt cells. Accordingly, several studies on cultured intestinal epithelial cells and many other cell types have revealed a close correlation between ERK activation and DNA synthesis while pharmacological or molecular inhibition of ERK activity has been shown to block cell cycle progression [15,25]. Notably, like E2F4 [9], phosphorylated and activated forms of ERK1/2 have been mostly detected in the nucleus of undifferentiated proliferative crypt cells in human fetal small intestine [18], hence supporting the role of these kinases in cell cycle control of intestinal crypt cells. Therefore, our results indicate that one of the mechanisms by which ERK1/2 MAP Kinases induce intestinal epithelial proliferation may be by promoting E2F4 nuclear translocation. Once into the nucleus, E2F4 may control the expression of proteins necessary for entry into S phase including *cdc6, dihydrofolate reductase (dhfr), thymidine kinase, cyclin E, cyclin A, mcm3* and *DNA polymerase α* as we have previously shown [2,10].

The exact molecular mechanism by which ERK1/2 promotes E2F4 nuclear translocation however remains unclear. Herein, E2F4 was found to be rapidly phosphorylated on serine residue(s) in serum-treated cells and that this phosphorylation was MEK-dependent. Of importance, we observed a strong correlation between the rapid phosphorylation of E2F4 and its subsequent nuclear translocation. Hence, one could speculate that phosphorylation of E2F4 by ERK1/2 is necessary to induce its translocation into the nucleus. However, although E2F4 phosphorylation was observed within 30 minutes after serum addition, nuclear accumulation of E2F4 only began after 4 hours [9]. This suggests that ERK-dependent E2F4 phosphorylation may represent an initiating event for nuclear translocation and that other mechanisms are also likely implicated. A possible mechanism could be the heterodimerization of E2F4 with its transcriptional partner DP-2, reported to promote E2F4 nuclear localization and activation [26]. One might speculate that E2F4 phosphorylation could promote its association with DP-2 and subsequently, its nuclear re-localization. To our knowledge, few studies have demonstrated phosphorylation of E2F4 [27-30], and none have linked phosphorylation to the stimulation of E2F4 function or transcriptional activity. We show herein that ERK kinases can efficiently and directly phosphorylate E2F4 protein *in vitro*, thus identifying E2F4 as a novel target of ERK kinases, adding to the list of ERK substrates implicated in cell proliferation control [31]. Our analysis of putative ERK1/2 phosphorylation sequences revealed that S244 and S384 were both implicated in the transcriptional activity of E2F4 and in its nuclear localization. Nevertheless, more studies are needed to clearly elucidate the molecular mechanisms of E2F4 activity and localization by phosphorylation.

A novel and intriguing finding of this study is that stimulation with EGF was not sufficient to induce G1/S phase transition of human non immortalized intestinal epithelial cells. These results contrast with those observed in rodent immortalized intestinal epithelial cell lines in which EGF induced DNA replication and proliferation [15]. Furthermore, many studies have reported proliferative properties of EGF in organotypic cultures of human fetal intestinal epithelium, although these studies did not exclude the contribution of other mesenchymal and epithelial factors in this effect [32-34]. Many studies from our laboratory and others have clearly demonstrated that HIEC are useful and relevant in analyzing the regulation of proliferation of intestinal epithelial crypt cells in humans [9,10,35,36]. Indeed, the expression of intestinal epithelial-specific keratins [37], of components specific to cell junctions [38], cell cycle-related proteins [9,10,39], as well as typical intestinal cell markers of undifferentiated lower crypt cells [37,39,40] indicate that these cells behave as cells representative of the bottom of the human crypt [41]. Their epithelial cryptal origin was also confirmed by their ability to express the 350-kD crypt cell-specific marker MIM-1/39 [42]. Interestingly, we show herein that although EGF induced a rapid activation of ERK1/2 similarly to serum and LPA, this action was not sufficient for S phase entry as visualized by the absence of pRb hyperphosphorylation, cyclin A protein expression and E2F4 nuclear translocation. In addition, EGF did not trigger the degradation of the cell cycle inhibitor p27, an event necessary to exit the quiescent state and pass the restriction point [15]. The failure of EGF to induce G1/S transition in HIEC could be explained by its inability to promote a sustained phosphorylation of Akt. Indeed, stimulation of Akt by growth factors is known to be required for G1 progression and S phase entry of many cell types [43] including intestinal epithelial cells [16]. Several hypotheses could be suggested to explain why EGF alone is not sufficient to promote a strong and sustained Akt activation in HIEC. First, the nature of EGFR-associated Ras proteins, i.e. H-Ras or K-Ras, can define the selective activation of ERK or Akt pathway by EGF [44]. In addition, in colon cancer, activation of the Rho/Rho-kinase pathway inhibits the capacity of EGF to promote Akt activation, but not ERK1/2 [45]. Finally, Erk5 was recently shown to be necessary for sustained PDGF-induced Akt phosphorylation in endothelial cells [46]. Further studies are thus needed to verify whether these different hypotheses could explain why EGF alone did not promote a sustained Akt activation in human intestinal epithelial cells.

Our results also suggest that phosphorylation and inhibition of GSK3β play an important role in the nuclear translocation of E2F4 and the proliferative response of HIEC. Indeed, serum and LPA, but not EGF, inactivated GSK3β as visualized by the sustained phosphorylation on serine 9, probably triggered by Akt or protein kinase C [22]. Furthermore, when GSK3β was pharmacologically inhibited, EGF induced pRb hyperphosphorylation, cyclin D1 expression, p27 degradation and E2F4 nuclear translocation, four events associated with G1/S phase transition. It is noteworthy however, that inhibition of GSK3 only partially rescued the inability of EGF to induce E2F4 nuclear translocation and cell proliferation in comparison to serum. This could be explained by the fact that serum contains a number of different additional factors (including LPA) and hormones in high concentrations that may activate various signaling pathways including calcium mobilization and PKCs, other signaling events known to promote proliferation [47]. Nevertheless, our results suggest that GSK3β, which is tonically active in quiescent cells, must be phosphorylated and inactivated to enable cell cycle progression of HIEC. GSK3β im-plication in E2F4 nuclear localization control adds to the previously described role of GSK3β on E2F1 regulation by ubiquitination and degradation resulting in a reduced transcriptional activity [48]. This is also reminiscent of the observed decreased expression of several other cell cycle regulated proteins following GSK3 activation, including c-myc, cyclin D1 and β-catenin [22]. In this regard, accumulation of these GSK3 substrates has been linked to increased intestinal proliferation and notably frequently observed in colorectal cancers [20,23,49,50].

## Conclusion

E2F4 protein expression is up-regulated in human colorectal cancers [10,51]. Accordingly, we have previously shown that E2F4 expression is necessary for both anchorage-dependent and independent growth of colorectal cancer cells [10]. Results of the present study demonstrate that E2F4 protein levels were significantly enhanced in human adenomas, at an early stage of colorectal cancer. Interestingly, E2F4 expression was mostly detected in the nucleus and appeared to be phosphorylated in adenomas. Of note, the MEK/ERK and GSK3 signaling pathways, shown herein to be implicated in the regulation of E2F4 phosphorylation and localization, are known to be involved in colorectal adenoma formation [23]. Hence, our findings suggest that dysregulated E2F4 nuclear localization may represent one of the instigating events leading to hyperproliferation and hence of tumor initiation and promotion in the colon and rectum.

## Methods

### Material and antibodies

MEK inhibitors U0126 and PD184352 were purchased from LC Laboratories (Woburn, MA, USA) and GSK3 inhibitor SB216763 was purchased from Sigma-Aldrich (Oakville, ON, Canada). Antibodies against E2F4 (C-20), cyclin D1 (C-17), cyclin A (H-432), p27 (C-19), ERK1 (C-16), ERK2 (C-14) and HA-probe (F-7) were all purchased from Santa Cruz Biotechnology Inc. (Santa Cruz, CA, USA). Phospho-ERK1/2 (Thr202/Tyr204) #9101, phospho-GSK3β (Ser9) #9336, phospho-glycogen synthase (GS) (Ser641) #3891, phospho-ERK5 (Thr218/Tyr220) #3371 and phospho-Akt (Ser473) #9271 were from Cell Signalling Technology (Danvers, MA, USA). β-actin monoclonal antibody (clone C-4) and recombinant active ERK1 were obtained from Millipore (Billerica, MA, USA). pRb and Ki67 monoclonal antibodies (clone 35) were purchased from BD BioSciences (Mississauga, ON, Canada) and phospho-serine antibody (clone 16B4) from BIOMOL International (#SA-337). Alexa Fluor® 488 goat anti-rabbit and Alexa Fluor® 568 goat anti-mouse antibodies were from Invitrogen (Carlsbad, CA, USA). [γ-^32^P]ATP was obtained from PerkinElmer (Waltham, QC, Canada). PP1 phosphatase was obtained from New England Biolabs Ltd. (Pickering, ON, Canada). All other materials were from Sigma-Aldrich (Oakville, ON, Canada) unless stated otherwise.

### Expression vectors and construct

The expression vector (pCMV) for HA-DP-2 was obtained from Dr. J.A. Lees (Department of Biology, Massachusetts Institute of Technology, Cambridge, USA). The expression vector (pCDNAneo3) for E2F4 was obtained from Dr. C. Sardet [52] (Institut de Génétique Moléculaire, Montpellier, France). The full-length human E2F4 cDNA was subcloned in expression vector pCDNA3.1 in frame with the HA-tag (at E2F4 N-terminal end). PCR were performed to insert the HA tag in the E2F4 sequence using oligonucleotides containing the HA-tag and a KOZAK sequence: 5′ – A GAC TAG GAT CC C ACC ATG TAT GAT GTT CCT GAT TAT GCT AGC CTC CCG GCG GAG GCC GGG CCA CAG GCG CCG – 3′ and 5′– TCT GTA CTC GAG TCA GAG GTT GAG AAC AGG CAC ATC AAA GAG GTC – 3′. PCR products were next ligated in BamHI/EcoRI-digested pCDNA3.1 vector. The E2F4 mutants T224A and T248A were generated by site-directed mutagenesis using the following oligonucleotides, respectively: 5′-CCA TCT GCT GTT TCG GCG CCT CCA CCT CTG CCC AAG- 3′ and 5′-CTT GGG CAG AGG TGG AGG CGC CGA AAC AGC AGA TGG- 3′; 5′-AAT AGT CCT CAG CTA GCG CCC ACT GCT GTC CCT- 3′ and 5′- AGG GAC AGC AGT GGG CGC TAG CTG AGG ACT ATT- 3′. PCR products were next digested and ligated in BamHI/EcoRI-digested pCDNA3.1 vector. E2F4 mutants T14A, S202A, S218A, S244A, S384A were generated by Genscript (Piscataway, NJ, USA). E2F4 mutants S244E and S384A were generated by site-directed mutagenesis using the following oligonucleotides, respectively: 5′ –TCACGTCCAAATGAACCTCAGCTCACT – 3′ and 5′ – AGTGAGCTGAGGTTCATTTGGACGTGA – 3′; 5′ – TGCCCCTCTGCTTCGTCTTGAACCAC – 3′ and 5′ – GTGGTTCAAGACGAAGCAGAGGGGCA – 3′.

### Cell culture

Non-Immortalized Human Intestinal Epithelial Cells (HIEC) were isolated by Perreault and Beaulieu [37] from normal human fetal intestinal epithelium at mid-gestation. Cells were cultured in Opti-MEM (Invitrogen, Burlington, ON, Canada) supplemented with 2 mM glutamine (Invitrogen), 5% Fetal Bovine Serum (FBS), 10 mM HEPES, 0.5 IU/ml penicillin, 50 μg/ml streptomycin (all obtained from Wisent, St-Bruno, Canada) and 0.2 IU/ml insulin (Connaught Novo Laboratories, Willowdale, ON, Canada). These cells express typical features of the lower adult crypt region [37,39,40] and are unable to differentiate. The life span of these normal non immortalized cells is limited to 22–25 passages. Human Embryonic Kidney 293T cells (ATCC, Manassas, VA, USA) were cultured in Dulbecco′s Modified Eagle’s Medium (DMEM; Invitrogen) containing 10% FBS supplemented with 2 mM glutamine, 10 mM HEPES, 0.5 IU/ml penicillin and 50 μg/ml streptomycin.

### HIEC synchronization experiments

HIEC (p18-p21) were grown to a density of 70-80% and were serum-deprived for 36 h in DMEM after two washes with Phosphate Buffered Saline (PBS) and two washes with DMEM medium. FACS analysis confirmed that 99% of cells were quiescent and in G0 (unpublished data). Cells were then stimulated with 5% FBS, 100 ng/ml EGF or 10 μM LPA for 30 min or 24 h with or without a 10 min pre-treatment with DMSO, MEK inhibitors U0126 (20 μM) or PD184352 (5 μM) or GSK3 inhibitor SB216763 (20 μM).

### Protein extraction and immunoblotting

Cells were washed twice with ice-cold PBS then lysed in Triton lysis buffer (1% Triton X-100, 50 mM Tris–HCl pH 7.5, 100 mM NaCl, 5 mM EDTA, 5% glycerol, 40 mM β-glycerophosphate, 50 mM NaF, 200 μM orthovanadate, 5% pepstatin, 5% aprotinin, 5% leupeptin and 5% phenylmethylsulfonyl fluoride (PMSF)) for 30 min under light agitation. Lysates were then cleared by centrifugation (15 000 *g*, 10 min) and 4X Laemmli buffer (2.3% SDS, 10% glycerol, 0.005% bromophenol blue and 5% β-mercaptoethanol) was added to supernatants for gel analysis. Whole cell extracts were separated on 7.5% (for E2F4 shift-up) or 10% SDS-PAGE gels and then electro-transferred onto polyvinylidene fluoride (PVDF) membranes (Perkin Elmer). Membranes were blocked for 1 h at 20°C using 0.05% Tween/PBS containing 5% non-fat dry milk then incubated overnight in primary antibodies diluted in blocking solution. Membranes were next incubated with horseradish peroxidase-conjugated goat anti-mouse or anti-rabbit IgG (GE Healthcare, Baie d’Urfé, QC, Canada) in blocking solution for 1 h. The blots were visualized using homemade ECL (Tris–HCl 100 mM pH8.5, 1.25 mM luminol, 225 μM coumaric acid and 2.9 mM H_2_O_2_). Protein concentrations were measured using BCA procedure (ThermoScientific, Waltham, MA, USA) as described by the manufacturer with bovine serum albumin (BSA) as standard.

### Immunofluorescence

HIEC were grown on glass coverslips to 70-80% confluency. Cells on coverslips were rapidly rinsed with PBS and then fixed with 3% paraformaldehyde/PBS for 20 min. Cells were permeabilized with 0.1% Triton X-100/PBS for 10 min and blocked with 2% BSA/PBS for 20 min. Cells were incubated for 2 h with primary antibodies diluted in blocking solution then immunostained with anti-rabbit AlexaFluor 488 and anti-mouse AlexaFluor 568 conjugated secondary antibodies. For each experiment, negative controls (no primary antibody) were included. Additional controls were performed to ensure the absence of cross-reactivity between the wavelengths. Cells were incubated with Ki67 primary antibody after which AlexaFluor 568-coupled secondary antibody was added and fluorescence observed at the designated wavelengths used for E2F4 observation (AlexaFluor 488).

### PP1 phosphatase assay

HIEC were serum-starved for 36 h then stimulated for 30 min with 5% FBS. Cells were lysed in Triton lysis buffer without phosphatase inhibitors. Cleared lysates were incubated with anti-E2F4 antibody (3 h, 4°C) after which protein A Sepharose CL-4B beads (GE Healthcare) was added for an additional hour. Immunocomplexes were washed four times with Triton lysis buffer without phosphatase inhibitors and twice with phosphatase assay buffer provided by the manufacturer. Thereafter, 2.5 units of PP1 were added to E2F4 immunocomplexes in phosphatase buffer and incubated at 30°C for 30 min. Laemmli’s buffer was added to stop the reaction and samples were boiled and subsequently loaded on SDS-PAGE.

### Kinase assays

293T cells were transfected with pCDNA3.1 empty vector or pCDNA3.1 HA-tagged-wild-type E2F4 or mutants using Lipofectamine 2000 (Invitrogen) according to the recommended manufacturer protocol. Cells were lysed in Triton lysis buffer 48 h after transfection. To immunoprecipitate E2F4, cleared lysates (1 mg) were pre-incubated with HA antibody (3 h, 4°C) to which protein A Sepharose CL-4B beads were added for an additional hour. Immunocomplexes were washed four times with Triton lysis buffer and twice with kinase assay buffer (25 mM Tris–HCl pH 7.5, 20 μM EGTA, 10 mM β-glycerophosphate, 1 mM orthovanadate, 400 μM DTT, 30 mM MgCl_2_ and 30 mM BSA). [γ-^32^P]ATP (3 μCi/assay) was added and the reaction initiated by the addition of recombinant active ERK1 (5 ng) and incubation at 30°C. After 5 min, reactions were stopped with Laemmli’s buffer. Samples were boiled and radiolabeled E2F4 was separated from antibodies on SDS-PAGE gels. Results were visualized by autoradiography. After radiography, gels were transferred onto PVDF membranes and immunoblotted with HA antibody.

### Luciferase assays

293T cells were seeded in 6-well plates and co-transfected by lipofection (Lipofectamine 2000, Invitrogen) with 0.1 μg of thymidine kinase-luciferase reporter, 0.25 μg of the relevant expression vector (pcDNAneo 3) containing wild-type E2F4 or mutants and 0.25 μg of the relevant expression vector containing DP-2. The pRL-SV40 *Renilla* luciferase reporter vector was from Promega (Nepean, ON, Canada). Two days after transfection, luciferase activity was measured as previously described [9], according to the Promega protocol.

### Human adenomas

Samples of colorectal adenomas and paired normal tissues (at least 10 cm from the adenoma) were obtained from patients undergoing surgical resection. Patients did not receive neoadjuvant therapy. Tissues were obtained after patient’s written informed consent, according to the protocol approved by the Institutional Human Subject Review Board of the Centre Hospitalier Universitaire de Sherbrooke. All tissues were frozen in liquid nitrogen within 30 min from resection. Tissues were embedded, cryosectioned and immunostained, as described previously [10]. Genomic DNA was extracted from formalin-fixed paraffin-embedded tissue using a FFPE DNA Isolation Kit for Cells and Tissues (Qiagen). *APC* (exon 15), *KRAS* (exons 1 and 2) and *BRAF* (exon15) were amplified by PCR and the presence of mutations was detected by direct sequencing (Plateforme de Séquençage et de Génotypage des Génomes du CRCHUL, QC, Canada). Paired tissues were lysed in Triton lysis buffer and immunoblotted as described above.

### Data presentation

Assays were performed in triplicate. Typical Western blots shown are representative of three independent experiments. Representative results of *in situ* indirect immunofluorescence from three independent experiments are shown.

## Competing interests

The authors declare that they have no competing interests.

## Authors’ contributions

MCP performed the molecular and cellular studies, participated in the study design and drafted the manuscript. SC and CL participated to the molecular studies and to the critical reading of the manuscript. JCC participated in the study design and is the leader of the colorectal cancer tissues biobank. NR conceived the study, and participated in its design and coordination and helped draft the manuscript. All authors read and approved the final manuscript.

## Supplementary Material

Additional file 1: Figure S1 Subconfluent HIEC were serum-deprived for 36 h, treated or not (DMSO) during 10 min with the MEK inhibitors U0126 (20 μM) or PD184352 (5 μM) and then stimulated with 100 ng/ml EGF for 30 min. Thereafter, cells were lysed and proteins were analyzed by SDS-PAGE for Western blot analysis for the expression of phosphorylated ERK5, phosphorylated AKT, total AKT and β-actin. Click here for file

Additional file 2: Figure S2 Subconfluent HIEC were serum-deprived for 36 h, treated or not (DMSO) during 10 min with the MEK inhibitors U0126 (20 μM) or PD184352 (5 μM) and then stimulated with 10% serum for 30 min. Thereafter, cells were lysed and proteins were analyzed by SDS-PAGE for Western blot analysis for the expression of E2F4, ERK2, phosphorylated ERK2 and β-actin. Representative of three experiments. Click here for file
