# Supplementary material for: ERK-associated changes in E2F4 phosphorylation, localization and transcriptional activity during mitogenic stimulation in human intestinal epithelial crypt cells
Source: BMC Cell Biol. 2013 Aug 6;14:33. doi: 10.1186/1471-2121-14-33 (PMC3750237; doi:10.1186/1471-2121-14-33)
Supplement: Additional file 2: Figure S2 — Subconfluent HIEC were serum-deprived for 36 h, treated or not (DMSO) during 10 min with the MEK inhibitors U0126 (20 μM) or PD184352 (5 μM) and then stimulated with 10% serum for 30 min. Thereafter, cells were lysed and proteins were analyzed by SDS-PAGE for Western blot analysis for the expression of E2F4, ERK2, phosphorylated ERK2 and β-actin. Representative of three experiments. [file 1471-2121-14-33-S2.pdf]

Figure S2

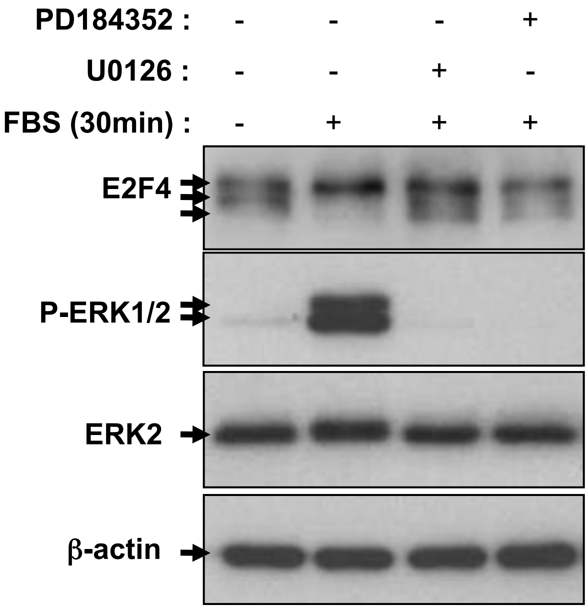

Subconfluent HIEC were serum-deprived for 36 h, treated or not (DMSO) during 10 min with the MEK inhibitors U0126 (20 μM) or PD184352 (5 μM) and then stimulated with 10% serum for 30 min. Thereafter, cells were lysed and proteins were analyzed by SDS-PAGE for Western blot analysis for the expression of E2F4, ERK2, phosphorylated ERK2 and β-actin. Representative of three experiments.
